# Binocular Coordination: Reading Stereoscopic Sentences in Depth

**DOI:** 10.1371/journal.pone.0035608

**Published:** 2012-04-27

**Authors:** Elizabeth R. Schotter, Hazel I. Blythe, Julie A. Kirkby, Keith Rayner, Nicolas S. Holliman, Simon P. Liversedge

**Affiliations:** 1 Department of Psychology, University of California San Diego, La Jolla, California, United States of America; 2 Department of Psychology, University of Southampton, Southampton, United Kingdom; 3 Department of Psychology, Bournemouth University, Bournemouth, United Kingdom; 4 School of Engineering and Computing Science, University of Durham, Durham, United Kingdom; University of Leicester, United Kingdom

## Abstract

The present study employs a stereoscopic manipulation to present sentences in three dimensions to subjects as they read for comprehension. Subjects read sentences with (a) no depth cues, (b) a monocular depth cue that implied the sentence loomed out of the screen (i.e., increasing retinal size), (c) congruent monocular and binocular (retinal disparity) depth cues (i.e., both implied the sentence loomed out of the screen) and (d) incongruent monocular and binocular depth cues (i.e., the monocular cue implied the sentence loomed out of the screen and the binocular cue implied it receded behind the screen). Reading efficiency was mostly unaffected, suggesting that reading in three dimensions is similar to reading in two dimensions. Importantly, fixation disparity was driven by retinal disparity; fixations were significantly more crossed as readers progressed through the sentence in the congruent condition and significantly more uncrossed in the incongruent condition. We conclude that disparity depth cues are used on-line to drive binocular coordination during reading.

## Introduction

While reading, our eyes make coordinated sideways movements (*saccades*) to bring the next part of the text into the fovea so that it may be processed with the highest resolution [1]–[2]. In the real world (i.e., not in a laboratory setting), we often encounter situations in which the text appears at different distances from us as we read it. That is, text exists in three dimensions with, for example, the word at the beginning of a sentence being further away from us than the word at the end of a sentence. Consequently, when we read such “looming” text, in addition to saccades, we must make *vergence* eye movements to accommodate changes in depth. The question, then, is how effective is the eye movement system in coordinating the eyes to make both saccades and vergence movements concurrently to ensure that that reading can proceed normally? Furthermore, what information does the system utilize in order to do so?

Considerable progress has been made over the past 40 years in our understanding of the way eye movements relate to the visual, cognitive, and linguistic processes involved in reading [1]–[3]. The vast majority of this knowledge, however, has been gained from studies when only one eye is monitored with the implicit assumption that the two eyes fixate the same location. Recently, however, the extent to which the two eyes are coordinated during reading has been directly examined [4]–[10] and it is quite clear that they do not always fixate the same letter; the two points of fixation are more than one character space apart on approximately half of the fixations (see Kirkby et al. [7], for a review). This work is consistent with an earlier literature in which binocular coordination during fixations and saccades, and the complex interactions between the vergence and accommodation systems in response to depth cues, have been well-documented [11]–[20]. It is quite clear that the eye movement system is able to deal with a certain amount of *fixation disparity* (offset between the locations of gaze of the two eyes) when reading, but what about disparity in more extreme circumstances?

All studies of eye movement control during reading to date, including those mentioned above, have investigated reading when the plane of the text is perpendicular to the line of sight (i.e., viewed in two dimensions). From research that has investigated binocular coordination during reading, it is clear that readers do not experience diplopia (double vision) and are able to read quite easily, despite the fact that we only fixate the same letter half the time. Liversedge, Rayner et al. [8] argued that this is achieved via fusion of the two inputs into a unified percept. Not all sentences appear perfectly perpendicular to the reader, however. To illustrate this point, imagine standing to the side of a billboard or road sign so that the beginning part of the text is further away from you than the end. Under such circumstances, we must not only make the necessary conjugate, sideways saccadic eye movements to bring the next word to the fovea but also disconjugate vergence eye movements to allow for the fact that fixations at the end of the text will be closer to us than those at the beginning. It is quite surprising that no studies to date have investigated binocular coordination in such situations, given that vergence movements could create more variability in fixation disparity, and consequently might impact the reader’s ability to fuse the binocular signal into a single percept. Thus, we cannot assume that monocularly measured reading behavior observed when text is presented perpendicular to the reader’s line of sight, and which is so well understood, would necessarily be the same as that observed when the text requires the reader to make substantive vergence movements in addition to saccadic movements in order to read. Note also, that vergence movements in response to more simple visual stimuli, such as in response to simple dot stimuli, are fairly well understood [21]. However, as indicated above, it is far less clear what drives vergence movements when sentences are read that appear in three dimensions.

Vergence responses ordinarily occur quite rapidly, especially following saccades [22]–[23] and are mostly driven by foveal retinal disparity [24]. Blythe, Liversedge, and Findlay [25] found that, for visual linguistic stimuli (words and nonwords), the oculomotor system is differentially responsive to stereoscopic disparity cues in the fovea and parafovea. Blythe et al. measured binocular eye movements while subjects made a saccade onto a word presented 1.3° away from fixation (in the parafovea) with varying magnitudes of stereoscopic disparity (0-, 1- or 2-character disparities). The disparity was either crossed (the right eye image was displaced to the left of the left eye image) so that the word appeared in front of the screen, or uncrossed (the right eye image was displaced to the right of the left eye image) so that the word appeared behind the screen. Blythe et al. found that vergence during initial saccades onto the word was minimally affected by the magnitude or direction of the stereoscopic manipulation. Importantly, however, during initial fixation on the word, vergence was highly responsive to apparent depth induced by stereoscopic offset. Readers responded to words with crossed stereoscopic disparity by making convergent movements and responded to words with uncrossed stereoscopic disparity by making divergent movements. These results suggest that the oculomotor system is insensitive or unresponsive to stereoscopic disparity cues associated with words in the parafovea, but very responsive in terms of vergence movements when those words are directly fixated.

In contrast to Blythe et al.’s results, there is evidence that, with practice, subjects can improve vergence responses to parafoveal stereoscopic depth cues. Eggert and Kapoula [26] asked subjects to saccade repeatedly between two points in a stereoscopic display for 15 minutes. Although appropriate vergence during saccadic targeting was initially poor, towards the end of this prolonged test period it improved such that the eyes realigned appropriately in depth during the saccade. Taken together, the results of these two studies suggest that, ordinarily, the oculomotor system is initially unresponsive to stereoscopic disparity cues in the parafovea, but this initial lack of response can be overcome with oculomotor rehearsal.

While the Blythe et al. [25] and Eggert and Kapoula [26] studies (amongst others) represent important foundations of our current understanding of basic binocular oculomotor control, the studies used single words or point light sources as stimuli. Neither study investigated binocular coordination during normal reading. Reading is a complicated process that involves the precise coordination of psychological systems associated with vision, oculomotor control, and linguistic processing in real time. How the eyes are coordinated binocularly concurrently with such processing is a complex issue; it is not clear that the effects observed in simpler tasks will necessarily generalize to eye movement behavior associated with normal reading in three dimensions.

The locations of the fixations that we make when we move our eyes through text during reading are not random. Instead, readers target their saccades fairly precisely based on information that is obtained from the parafovea [27]. Experiments investigating saccadic targeting in reading (measuring eye movements from one eye) have established that saccades are targeted to just left of the center of words (the *Preferred Viewing Location*, *PVL* [28]). However, one study that examined binocular saccadic targeting during normal reading utilized a dichoptic presentation method in which a different stimulus was presented to each eye [8]. Specifically, target compound words such as *cowboy* were embedded in the sentences. While the rest of the sentence frame was presented binocularly, the target words were presented dichoptically such that one portion of the word was presented to the left eye only (e.g., *cowb*) and the other portion of the word was presented to the right eye only (e.g., *wboy*). In a control condition, the entire sentence and the entire target word were presented binocularly. Three outcomes were possible: (a) If each eye were making a saccade based on its own retinal input, the eyes would land in different locations (i.e., targeting the PVLs of their respective part-word stimuli), (b) If saccades were programmed based on the input of one eye then both eyes would land on the same location (i.e., the PVL of one of the two part-word stimuli), (c) If saccades were programmed based on a single, unified percept landing positions would reflect the PVL of the entire word. Saccade targeting based on a unified percept is exactly what Liversedge et al. [8] found: landing position distributions of the two eyes on these target words corresponded to the PVL of the entire word, even in the dichoptic presentation conditions. These data suggest that, while reading, subjects are able to (a) obtain information in the parafovea (b) fuse that information into a unified percept and (c) plan saccades based on that percept. Again, however, Liversedge et al. [8] only examined reading in two dimensions and ignored the fact that readers often need to make vergence movements as they fixate a sentence that changes in depth.

In the Blythe et al. [25] and Eggert and Kapoula [26] studies, the stimuli were spatially separated, with uniform disparity across the whole stimulus. Also, the dichoptic word part stimuli that Liversedge, et al. [8] used were perfectly complementary and aligned such that there was no disparity (alignment offset) between the stimuli delivered to the left and right eye. Furthermore, sentences were displayed at the plane of the computer screen, perpendicular to the line of sight. It may be the case that sentences that appear to loom or recede from the screen continuously, that is with varied levels of disparity across the whole sentence, provide a richer disparity gradient cue, because disparity is instantiated at every point in the sentence in a predictable, linearly increasing manner. It is possible, that when a uniform disparity gradient is available, the vergence system might be more responsive to disparity cues initially, because they exist continuously throughout the stimulus. If this were the case, what drives any such vergence movements?

There are two classes of cue to depth that the visual system may use as a basis for vergence. First, monocular depth cues are those that are functional with just one eye, such as *retinal size*; a given object, when closer to the viewer, will cover more retinal area than the same object at a further distance. This is such a strong depth cue that, when combined with a conflicting cue (e.g., linear perspective), it can produce a strong visual illusion (e.g., the Ponzo illusion and the Ames room illusion). Second, binocular depth cues occur due to differences in the relative position of two similar images on the retinas of the two eyes (i.e., *retinal disparity*). The magnitude of offset between the location of a given object in one retinal image relative to the other provides an estimate of depth.

Several studies have investigated the perceptual consequences of non-linguistic stimuli with conflicting binocular and monocular depth cues (e.g., grid stimuli where perspective cues suggest that the grids slant in one direction while disparity cues suggest that the slant is in the opposite direction). Such stimuli can give rise to perceptual ambiguity, where the apparent slant of the stimulus alternates between that dictated by perspective and that dictated by disparity [29]–[30]. For stimuli with conflicting perspective and disparity cues, there is much inter-individual variation in the dominant percept [29], though subjects are able to select and maintain one of the two percepts when instructed to do so [30]. However, for fairly brief presentations (e.g., 0.1, 1, and 10 seconds) perspective information tends to dominate over conflicting disparity cues, but for longer presentations (30 seconds) the percept is increasingly dominated by disparity cues [31]. These findings raise the question of whether, during reading, vergence would be driven by monocular or binocular depth cues. On the basis that disparity depth cues dominate perception during extended rather than brief viewing durations, we might predict that during reading, disparity cues would drive vergence. To investigate this, we manipulated both monocular and binocular depth cues to assess which one is more deterministic of the vergence response.

In the present study, subjects were required to read for comprehension. Successful comprehension is only possible if the reader successfully fuses the two images (i.e., those projected to each eye separately) to produce a clear, single percept. Liversedge et al. [8] found that readers target saccades based on the unified percept (both eyes targeted the same location, even when different parts of the word were presented to the two eyes). Furthermore, Blythe et al. [25] found that the effective fusional range for subjects performing a lexical decision task was approximately one character space and, as disparities increased beyond this range, response times increased and accuracies decreased. These data indicate that, when fusion was not achieved, cognitive processing was adversely affected. Were the subjects able to suppress one output and base their lexical decision on the other, accuracies and RTs would have been unaffected for large disparities. Given these data, we are confident that subjects were fusing the images in our study because their comprehension of the sentences was not adversely affected by the stereoscopic manipulation and they did not report diplopia. If the reader is unable to fuse the image into a unified percept, then they experience diplopia (double vision).

**Figure 1:**
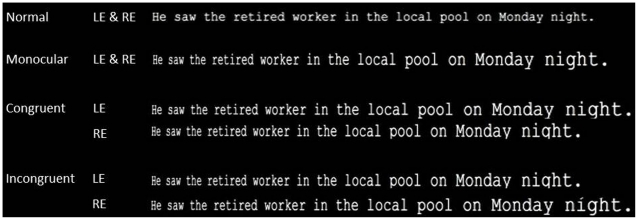
Examples of stimuli in the four experimental conditions: monocular cue, normal, congruent (retinal size and disparity cues for a looming sentence), and incongruent (retinal size cues for a looming sentence, disparity cues for a receding sentence).

We recorded the movements of each eye simultaneously to determine how the two eyes were coordinated in order to facilitate that fusion. Subjects read sentences that were presented stereoscopically (see Figure 1) in four different conditions: (a) *normal*, in which there were no depth cues, (b) *monocular cue*, in which retinal size was consistent with the text looming forwards from the screen, (c) *congruent*, in which both the monocular cue and retinal disparity was consistent with the text looming forwards from the screen and (d) *incongruent*, in which retinal size was consistent with the text looming forward from the screen, but disparity was consistent with the text receding behind the screen. Both the monocular and binocular depth cues increased in magnitude from the beginning to the end of the sentence so that the sentence appeared to extend increasingly closer or further from the plane of the screen as the subject read through it. As noted above, we recorded subjects’ binocular eye movements as they read sentences in these four conditions, to examine the behavioral response to congruent and incongruent disparity and monocular depth cues. The monocular cue condition was an important control condition as previous work has shown that fixation disparity exists for sentences presented in two dimensions, and is affected by the amplitude of the incoming saccade; the greater the amplitude of the incoming saccade, the greater the disparity observed at fixation onset [7]. Thus, the increasing font size across the sentence in this condition (corresponding to the monocular depth cue) would produce increasing saccade amplitudes and, thus, we anticipated increased fixation disparity through the sentence that did not necessarily reflect a response to perceived depth.

We made three predictions for this experiment. First, there would be a slight tendency for the eyes to be uncrossed in the normal and monocular cue condition and this effect would be larger in the monocular cue condition due to increasing saccade length, which is known to be correlated with larger uncrossed disparities [4], [6], [8]–[9]. Second, in the congruent condition fixation disparity would become increasingly crossed as subjects progressed from left to right through the sentence (as both monocular and disparity cues are consistent with the sentence looming towards the subject). Third, in the incongruent condition, there may be two possible outcomes: If monocular cues dominate and determine binocular coordination then fixation disparity ought to become increasingly crossed from left to right through the sentence. Alternatively, if binocular (disparity) cues dominate and primarily determine coordination then fixation disparity ought to become increasingly uncrossed from left to right, as this cue implies that the sentence recedes from the subject. Of these two hypotheses, the latter may be more likely due to an extended viewing period [31].

## Methods

### Ethics Statement

The investigation adhered to the principles of the Declaration of Helsinki, and was approved by the Ethics Committee at the School of Psychology, University of Southampton for human experimentation. Informed written consent was obtained from each subject after explanation of the procedure of the experiment.

### Subjects

Twelve subjects from the University of Southampton community participated in the experiment. Due to problems with the experimental equipment only a subset (four subjects) read sentences in the congruent condition. While the fact that there were fewer subjects in one condition is not ideal, the *linear mixed effects (lme) models* we used in the analyses (see Results section) are able to handle such missing data. The missing data in the congruent condition does not affect comparisons that do not involve the congruent condition and, in analyses that do involve the congruent condition, the concern is only about low power. Thus, one should only be concerned about real effects not yielding statistically significant results, as opposed to null effects yielding spurious significant results To provide further support for this argument, we performed the same analyses using only those subjects that experienced all conditions and the results corroborate those with our reported analysis of the full dataset. Subjects were between the ages of 22 and 35 and had normal or corrected-to-normal vision, and exhibited no reading problems. Subjects without functional stereopsis were excluded from participating in this experiment. Stereoacuity was assessed using the graded circle test. Subjects were judged to have functional stereopsis if they could correctly describe a stimulus with depth of 40 seconds of arc. Subjects were compensated £6 for every hour they took part in the experiment.

### Materials and Design

Each subject read 12 sentences in each of the different display conditions (explained above, see Figure 1): (a) *normal*, (b) *monocular cue*, (c) *congruent*, and (d) *incongruent*. These experimental conditions were presented in blocks, the order of which was counterbalanced between subjects. Sentences were counterbalanced across conditions so that each sentence was seen in every condition. In the normal condition, 3.42 characters equaled one degree of visual angle, in the other three conditions, in which there was a monocular depth cue of increasing retinal size, characters per degree ranged from 5.68 to 1.99.

All subjects bit on a wax dental mold and used forehead rests during the experiment, to eliminate head movements. Binocular eye movements were obtained from two Fourward Technologies Dual Purkinje Image eye trackers (one recording the movements of the left eye and the other recording the movements of the right eye, concurrently) and the position of each eye was recorded every millisecond. In all conditions, calibration routines and data collection were conducted using in-house software. The same software was used to present the images in all conditions.

The stereo sentence bitmaps were constructed using the OpenGL framework to render two separate images for the left and right eyes, where each image was drawn from a different camera perspective. First, the text in a particular sentence was rendered, with antialiasing, to a bitmap with no distortion. This bitmap was then used as a texture map applied to a rectangular quad element that was placed in OpenGL 3D space in such a way that one edge of the quad is nearer to the camera than the other (this gives the text the appearance of ‘looming’ towards the viewer). The asymmetric frustum parallel projection approach was used to setup the stereo camera projections. Information about the methods of this approach can be found at the following websites: http://www.orthostereo.com/geometryopengl.html; http://paulbourke.net/miscellaneous/stereographics/stereorender/The cameras were shifted apart by an intraocular distance of 65 cm and oriented in the same, parallel direction and the viewing frustums were adjusted in an asymmetric way to ensure zero disparity at the screen depth. This method was used to create stereo image pairs for each sentence.

Sentences were presented on a 21-inch Phillips CRT monitor positioned 100 cm from the subject. FE1 shutter goggles (Cambridge Research Systems), which alternate between left-open/right-shut and left-shut/right-open at the same refresh rate as the monitor (8 ms), were used to display the images stereoscopically [8], [25].

### Procedure

Subjects were first tested for stereopsis, then the two eye trackers were calibrated, and then the experimental trials began. For the initial calibration, and all checks of calibration accuracy, viewing was monocular – during calibration of the right eye tracker the left eye was occluded, and vice versa. The calibration was conducted using three points: one on the left of the screen, one on the right, and one in the center; the range of these three points was greater than the horizontal extent of the experimental stimuli. Once calibration had been completed, subjects were instructed to read the sentences normally for comprehension. During all conditions, subjects were instructed to maintain their fixation on the first word for several seconds until the experimenter told them to start reading; this allowed their percept to stabilize before beginning to read the sentence. This maintenance of fixation lasted approximately the same amount of time on each trial and was not determined by the subjects, themselves. Thus, it is not the case that this fixation indicated a requisite amount of time needed by the subjects to fuse the image, but was determined by the experimenter, irrespective of the subject’s behavioral response to disparity cues. Subjects then read the sentence and pressed a button when they had finished reading it. The button press terminated the trial. Subjects were instructed to read the sentences to the best of their ability. The accuracy of calibration was checked after every sentence. On a third of the trials, the sentence was immediately followed by a comprehension question (requiring a yes/no response). Each block took between 10 and 20 minutes to complete.

## Results

### Comprehension Accuracy

Subjects responded correctly to 77% of the comprehension questions (71% in the monocular cue only condition, 85% in the normal condition, 69% in the congruent condition and 81% in the incongruent condition), indicating that they read and understood the sentences. A logistic regression revealed that there were no significant differences between the monocular condition and the other conditions, except the normal condition produced marginally higher accuracy (z = 1.88, *p* = .06). No subject reported experiencing diplopia and all subjects reported experiencing the sentences in depth in the binocular depth cue conditions (this was expected because all subjects were screened to ensure that they had functional stereopsis).

### Data Processing Procedures

Fixations were manually identified in order to avoid contamination by dynamic overshoots [8], [32]. Initial fixations (i.e. those during which the subject was required to hold fixation on the first word in order to stabilize the percept) were excluded, as these were artificially long and were not “normal” reading fixations. Fixation durations between 80 and 1200 ms were considered valid and included in the analyses. These exclusion criteria resulted in 3805 fixations (95.7% of the data) that were used in the monocular reading measures. Additionally, for the binocular reading analyses, regressive (backward) saccades and fixations following them (28.1% of the data) were excluded from the analyses because these saccades led to a decrease in the magnitude of both the monocular and binocular depth cues (these experimental manipulations were based on a left-right progression through the sentences). Because the vergence response in such situations may not reflect the same response as that which occurred during normal, progressive saccades (during which the manipulated depth cues consistently increased as the reader progressed through each sentence), we excluded these cases. Disparity was calculated at the onset and offset of every valid fixation. Additionally, fixations and saccades with disparities that were more than 2.5 standard deviations from the mean for each subject (4.1% of the data) were excluded from the analyses. This additional exclusion criterion resulted in a dataset of 2578 fixations that were used for calculating the binocular measures (67.8% of the dataset used in the monocular measures).

All measures were analyzed with linear mixed effects (lme) models. For both types of analyses experimental condition (with the monocular cue condition as the baseline so that, in the conditions that also included a disparity manipulation, we could examine the effects of disparity above and beyond the monocular depth cue that was also present) was entered as a fixed effect and subjects and items were entered as crossed random effects. Additionally, for analyses of binocular measures, the following factors were entered as fixed effects. For fixation disparity: *distance from the beginning of the sentence* (because this corresponds to increasing stimulus disparity in the stereoscopic conditions) and *fixation duration* (because longer fixations might allow for more vergence). For saccade disparity: distance from the beginning of the sentence and *incoming saccade amplitude* (given that it is known to impact binocular coordination). All variables, aside from experimental condition, were centered so that the main effects were interpretable as the effect of the variable of interest at the average value of all other variables; interactions are not affected by centering.

### Monocular Reading Measures

We analyzed global reading measures to determine whether the different reading conditions affected the ease with which the text was processed (see Table 1). In general, reading difficulty is positively related to *mean fixation duration* (the average duration of each fixation on a sentence), *total sentence reading time* (the total time spent reading the sentence), the *number of fixations*, and the *number of regressions* (backward saccades) per sentence (for reviews, see [1]–[2]). For all reading measures, there were no significant differences between the monocular cue condition and the normal condition (all *p*s>.05). There were no significant differences between the monocular cue and the congruent condition (all *p*s>.05) except that there were fewer regressions in the congruent condition (2.98, SE = .34) than in the monocular cue condition (3.96, SE = .14; *t* = 2.51, *p*<.05). The incongruent condition led to longer average fixation durations (331ms, SE = 3.91) than in the monocular cue condition (303 ms, SE = 3.51; *t* = 3.65, *p*<.001). However, there were no significant differences between the monocular cue and incongruent conditions in total sentence reading time, number of fixations, or number of regressions (all *p*s>.05).

**Table 1:** Means and standard errors for monocular and binocular dependent measures in each of the four experimental conditions.

	Monocular cue	Normal	Congruent	Incongruent
**Monocular reading measures**				
Total sentence reading time (ms)	5114 (104)	5202 (84)	4755 (283)	5410 (75)
Number of fixations	12.25 (.30)	12.38 (.32)	11.58 (.52)	12.24 (.26)
Mean fixation duration (ms)	303 (4)	316 (3)	324 (10)	330 (4)
Number of regressive saccades	3.96 (.14)	3.64 (.17)	2.98 (.34)	4.00 (.14)
Saccade amplitude (degrees)	1.61 (.02)	1.64 (.02)	1.56 (.03)	1.61 (.03)
**Binocular reading measures**				
Start of fixation disparity (degrees)	−.19 (.02)	−.14 (.02)	.13 (.02)	−.66 (.01)
End of fixation disparity (degrees)	−.12 (.02)	−.08 (.02)	.27 (.02)	−.65 (.02)
Vergence during saccades (degrees)	−.51 (.02)	−.55 (.04)	−.45 (.06)	−.90 (.05)
Proportion of saccades exhibiting convergence	.25 (.01)	.27 (.02)	.30 (.01)	.16 (.01)
Vergence during fixations (degrees)	.57 (.03)	.55 (.03)	1.03 (.10)	.13 (.03)
Proportion of fixations exhibiting convergence	.79 (.01)	.73 (.01)	.85 (.01)	.48 (.01)

These data, along with the comprehension accuracy data, reported above, suggest that, in general, the presence of depth cues did not impact the subjects’ cognitive processing difficulty (i.e., the monocular cue, congruent and normal conditions were not fundamentally different, but note the lower power in the congruent condition). However, if the depth cues conflicted (e.g., in the incongruent condition), subjects spent longer on each fixation, but otherwise, reading proceeded fairly normally.

### Binocular Reading Measures

We examined disparity at both the start and end of fixations and vergence during saccades and fixations (for raw means see Table 1, for model outputs see Table 2). Negative values represent uncrossed disparity (relative to the depth of the screen, with the left eye fixating to the left of the right eye) whereas positive values represent crossed disparity (with the left eye fixating to the right of the right eye).

For completeness, and to facilitate comparisons to previous research, we also calculated absolute disparity and ran the same analyses. Absolute disparity at the start and end of fixations were, respectively, 0.29 and 0.26 in the monocular cue condition, 0.27 and 0.25 in the normal condition, 0.35 and 0.41 in the congruent condition and 0.69 and 0.68 in the incongruent condition. Importantly, the lme analyses perfectly replicated the analyses performed on the average, signed disparity data. In addition, for the normal condition, we calculated the percentage of fixations that were aligned, crossed and uncrossed. At the start of fixations, we found 71% of fixations to be aligned (the two eyes were within one character space (0.29 degrees of visual angle) of each other), 6% to be crossed and 32% to be uncrossed. At the end of fixations, we found 72%, 11% and 17%, respectively.

**Table 2:** The lme models for start and end of fixation disparity.

Predictor	Coefficient	Std. Error	*t* value
**Start of fixation disparity**			
Monocular cue (intercept)	−0.19	0.04	−4.81***
Normal	0.06	0.02	3.87***
Congruent	0.34	0.02	14.67***
Incongruent	−0.52	0.02	−33.29***
Distance from sentence beginning	−0.004	0.002	−1.88
Saccade amplitude	−0.009	0.01	−0.75
Normal×distance	−0.00006	0.003	0.00
Congruent×distance	0.05	0.004	11.45***
Incongruent×distance	−0.07	−0.003	−19.69***
Normal×saccade amplitude	−0.02	0.02	−1.30
Congruent×saccade amplitude	0.04	0.03	1.56
Incongruent×saccade amplitude	0.008	0.02	0.44
**End of fixation disparity**			
Monocular cue (intercept)	−0.11	0.04	−2.70
Normal	0.06	0.02	3.24***
Congruent	0.40	0.02	16.59***
Incongruent	−0.58	0.02	−35.34***
Distance from sentence beginning	0.002	0.002	1.13
Fixation duration	0.0001	0.00008	1.57
Normal×distance	0.003	0.003	−0.84
Congruent×distance	0.05	0.004	11.93***
Incongruent×distance	0.05	0.003	−18.99***
Normal×fixation duration	0.00002	0.0001	−0.18
Congruent×fixation duration	0.0003	0.0002	−1.69
Incongruent×fixation duration	0.00005	0.0001	0.50

All variables except for experimental condition were centered before being entered into the analysis.

* *p*<0.05;

** *p*<0.01;

*** *p*<0.005.

#### Start of fixation disparity

In the monocular cue condition the eyes tended to be uncrossed (−0.19°, significantly different from 0; *t* = 4.81, *p*<.001). There was less uncrossed disparity in the normal condition, which was significantly different from the monocular cue condition (−0.14°; *t* = 3.87, *p*<.001). This indicates that the presence of monocular cues to depth did result in a small increase in fixation disparity. This is likely due to the fact that words were increasing in size in the monocular cue condition and larger word objects allow for greater magnitude of disparity (in degrees) with the two points of fixation staying relatively near each other (in terms of characters). Therefore, on average, the system would be able to tolerate more physical disparity because the eyes would still fixate the same character, given its larger size (but see analysis of the interaction with distance from the beginning of the sentence, below). In the congruent condition, fixations were significantly more crossed compared to the monocular cue condition (0.13°, *t* = 14.67, *p*<.001), whereas in the incongruent condition disparity was significantly more uncrossed than the monocular cue condition (−0.66°; *t* = 33.29, *p*>.001).

The effect of distance from the beginning of the sentence was not significant in the monocular cue or normal conditions (both *p*s>.05). This may seem to contradict our claim that the increased size of the words in the monocular cue condition led to greater disparity, as word size increases in the monocular cue condition as one moves through the sentence. It is important to note, however, that words in the sentences were of variable lengths (e.g., between 1 and 10 characters long). Therefore, the degree to which words increase in size (i.e., degree of visual angle) is, to some extent, offset by variability in word length. Thus, a long word at the beginning of the sentence would subtend a larger degree of visual angle than a short word at the end of the sentence. This variability is likely the reason that, in the monocular condition compared to the normal condition, there is a significantly greater amount of uncrossed disparity, which is almost significantly (t = −1.88) modulated by distance from the beginning of the sentence.

**Figure 2:**
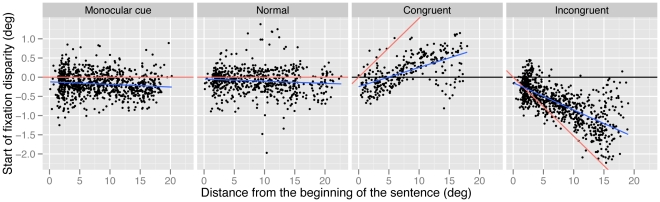
Disparity at the start of fixations, as a function of distance from the beginning of the sentence and experimental condition. Positive values denote crossed fixations (i.e., the eyes are aligned in front of the plane of the screen) and negative values denote uncrossed fixations (i.e., the eyes are aligned behind the plane of the screen). Black lines (set at 0°) represent the plane of the display screen; blue lines indicate the effect of distance from the beginning of the sentence on fixation disparity; red lines indicate the disparity that would be produced if subjects were to fuse the sentence as it appeared in virtual depth.

Most importantly, there was a significant effect of distance from the beginning of the sentence on fixation disparity in both the congruent (b = .05, *t* = 11.45, *p*<.001) and the incongruent condition (b = −.07, *t* = 19.69, *p*<.001); retinal disparity induced by the stereoscopic manipulation increased as distance from the beginning of the sentence increased. The direction of this disparity was opposite in the two conditions (see Figure 2); in the congruent condition, the magnitude of crossed fixation disparity increased as the reader progressed through the sentence, while in the incongruent condition the magnitude of uncrossed fixation disparity increased as the reader progressed through the sentence. There was no effect of incoming saccade amplitude on fixation disparity in any of the conditions (all *p*s>.5).

Overall, these data indicate that start of fixation disparity was overwhelmingly driven by stereoscopic disparity cues and not by monocular retinal size cues. Interestingly, as seen in Figure 2, the effects were very consistent within a condition. Virtually no crossed fixations were observed when stimuli were presented with uncrossed stereoscopic disparity, and vice versa. This indicates that all subjects were exhibiting a similar behavioral response to stereoscopic disparity in terms of binocular coordination, regardless of whether monocular cues were congruent or incongruent with the disparity cue. While we found some similar effects as those obtained under similar experimental testing conditions in the literature (i.e., the eyes tended to be uncrossed in the monocular cue and normal conditions [4], [6], [8]–[9], [33]), we did not find that the magnitude of disparity at the start of fixations was affected by the amplitude of the incoming saccade. This may seem puzzling, but one must bear in mind that saccade amplitude is correlated with the monocular depth cue because letters become stretched out as the sentence progresses from left to right. Additionally, the monocular depth cue (and consequently saccade amplitude) is correlated with the disparity manipulation because there is more stereoscopic disparity in the stimulus as the sentence progresses from left to right. Therefore, the lack of effect of saccade amplitude may be due to the fact that these other factors (with which it is correlated) are accounting for more variance in the data.

To test this, we conducted the same analysis with distance from the beginning of the sentence removed. This analysis revealed that saccade amplitude was related to start of fixation disparity in the monocular condition; longer saccades tended to produce more uncrossed fixation disparities (although the effect was marginal, *t* = −1.64, *p* = .11). There was no significant difference between the monocular and normal condition (*t*<1). In the congruent condition, the effect was significantly different from the monocular condition in the opposite direction–longer saccades produced more crossed fixation disparities, and in the incongruent condition the effect was larger than in the monocular condition and in the same direction–longer saccades produced more uncrossed fixations. Thus, we replicated the finding that larger saccade amplitudes produce greater disparity, but found that our manipulations of increasing retinal size and increasing retinal disparity were more deterministic of fixation disparity than saccade amplitude when both were entered in the analysis.

#### End of fixation disparity

**Figure 3:**
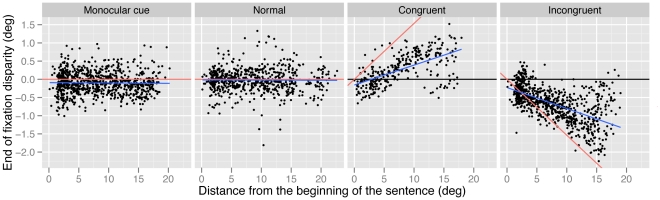
Disparity at the end of fixations, as the reader progresses through the sentence across the different experimental conditions. Positive values denote crossed fixations (i.e., the eyes are aligned in front of the plane of the screen) and negative values denote uncrossed fixations (i.e., the eyes are aligned behind the plane of the screen). Black lines (set at 0°) represent the plane of the display screen; blue lines indicate the effect of distance from the beginning of the sentence on fixation disparity; red lines indicate the disparity that would be produced if subjects were to fuse the sentence as it appeared in virtual depth.

The end of fixation data were broadly similar to the start of fixation data (see Table 2 and Figure 3). In the monocular cue condition the eyes tended to be uncrossed (−0.12°, significantly different from 0; *t* = 2.70, *p*<.01). In the normal condition, fixations tend to be significantly less uncrossed than in the monocular cue condition (−0.08°; *t* = 3.24, *p*<.005). As mentioned before, this slight increase in uncrossed fixations in the monocular condition compared to the normal condition is likely due to increased word size (see discussion of start of fixation disparity, above). The magnitude of fixation disparity was much greater in both stereoscopic conditions and the direction of this disparity was determined by the direction of the stereoscopic manipulation; fixations were more crossed in the congruent condition (0.27°; *t* = 16.59, *p*<.001) and more uncrossed in the incongruent condition (−0.65°; *t* = 35.34, *p*<.001) than in the monocular cue condition. Again, there were significant interactions with distance from the beginning of the sentence in both stereoscopic conditions, such that the magnitude of fixation disparity increased as the reader progressed through the sentence. In the congruent condition, fixations became more crossed as the reader progressed through the sentence (b = .05, *t* = 11.93, *p*<.001), while in the incongruent condition fixations became more uncrossed as the reader progressed through the sentence (b = .06, *t* = 18.99, *p*<.001). Additionally, fixation duration did not have a significant effect on end of fixation disparity (all *p*s>.5).

In summary, the end of fixation data were quite similar to the start of fixation data: in the monocular cue and normal conditions, the eyes tended to be slightly uncrossed. In the congruent condition the eyes tended to be crossed and in the incongruent condition the eyes tended to be more uncrossed than in the monocular cue condition. In the monocular cue and normal conditions the magnitude of the disparity was unaffected by position in the sentence while in the congruent and incongruent conditions the magnitude of this disparity increased as the subject progressed through the sentence. Therefore, it seems that fixation disparity was, for the most part, driven by the direction of stereoscopic disparity cues and very little by the monocular cue. Finally, end of fixation disparity was unaffected by fixation duration. As with the lack of an effect of saccade amplitude in the start of fixation disparity analyses, it is likely that the non-significant effect of fixation duration is due to the fact that stereoscopic disparity has such an influence on fixation disparity that the effects of other variables do not have the opportunity to exert an influence.

**Figure 4:**
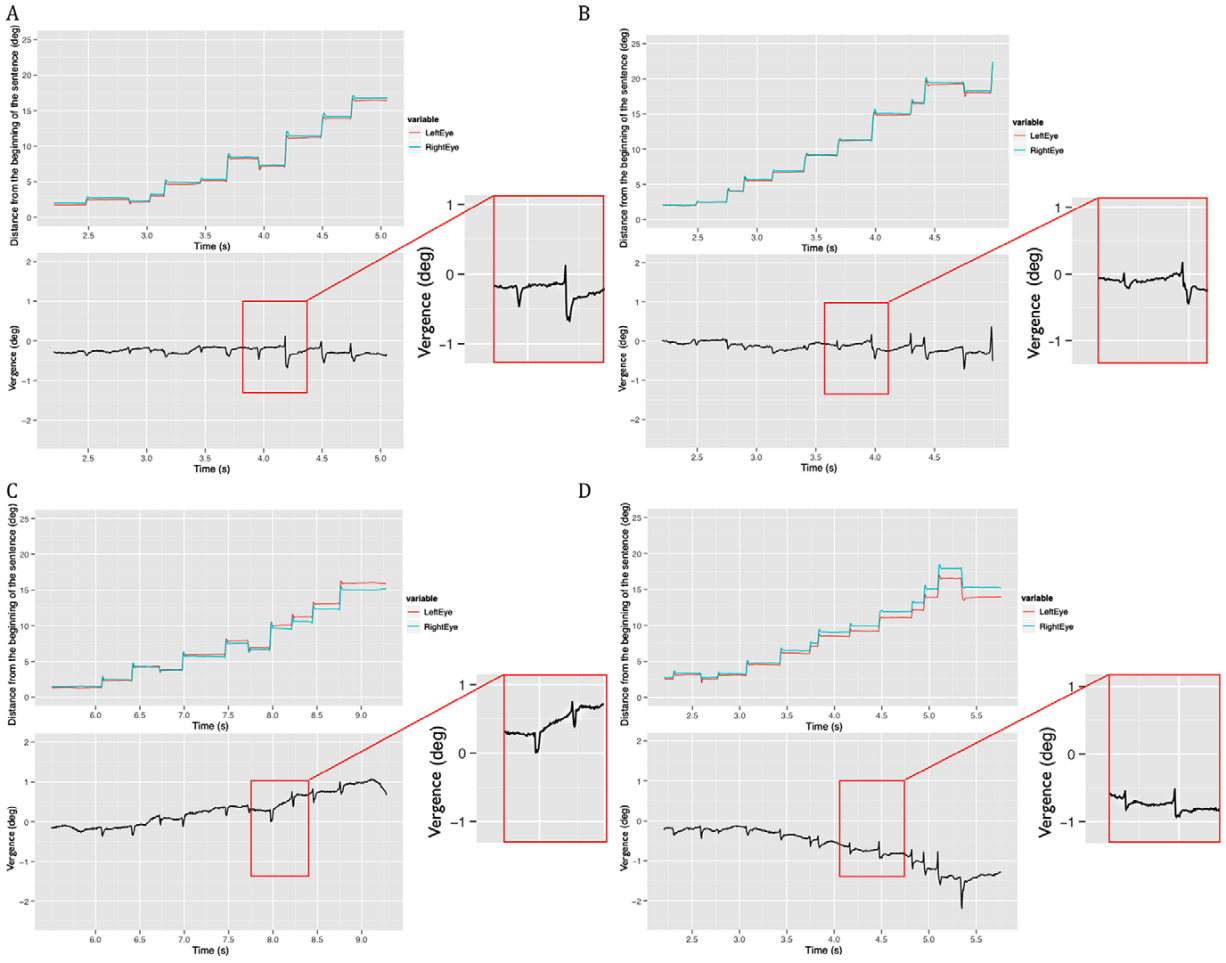
Left eye position, right eye position and disparity across time in single example trials in the different experimental conditions. In the top graphs, the red line represents the position of the left eye, the blue line represents the position of the right eye. The y axis represents distance from the beginning of the sentence and the x axis denotes time elapsed since the beginning of the trial (note that subjects were asked to hold fixation on the first word in the sentence for a few seconds; this time as been truncated and the plot represents only saccades and fixations associated with normal reading). In the bottom graph, the black line represents vergence of the two eyes; positive values denote convergence and negative values denote divergence. The insert enlarges the view of a series of two saccades. Panel A shows a trial in the monocular cue condition, panel B shows a trial in the normal condition, panel C shows a trial in the congruent condition and panel D shows a trial in the incongruent condition.

To illustrate the vergence responses that occurred during saccades and fixations across a trial, we plotted eye positions and vergence for example trials in each of the four conditions (see Figure 4). Formal analyses of the vergence response during fixations and saccades are reported in sections on vergence during saccades and vergence during fixations, below.

The panels in this figure clearly show that the eyes are quite well aligned to the same location on the screen in both the monocular cue and normal conditions. In contrast, in the congruent and incongruent conditions, where there is disparity in the stimulus, the eyes fixate somewhat different locations on the screen (consistent with the change in disparity in the stimulus; crossed in the congruent condition and uncrossed in the incongruent condition). Furthermore, these figures show that vergence occurs both during saccades and fixations. Fixations are represented by relatively stable values on the y-axis (location within the sentence) over changes in the x-axis (time). Saccades are represented by large changes in the y-axis during small increases in the x-axis. In the plots of vergence there is clearly a large transient change in disparity during the saccade (due to different and slightly unsynchronized changes in acceleration between the two eyes; see [11]). Apart from this transient change, the change in disparity from the end of the previous fixation to the beginning of the following fixation represents the amount of vergence observed during the saccade. In contrast, the slower change in disparity between the rapid, transient changes represents vergence during fixations. Change in disparity across the trial is most notable in the congruent and incongruent conditions and less so in the conditions without stereoscopic disparity manipulations.

#### Vergence during saccades

To evaluate the extent to which vergence occurred during saccades and fixations, we used linear mixed effects models to fit summarized trial-level data with the following dependent variables: *summed saccade vergence* (the sum of the vergence that occurred on every saccade in each trial), *proportion of saccade convergence* (the proportion of saccades on each trial where convergence as opposed to divergence was observed), *summed fixation vergence*, and *proportion of fixation convergence* (see Table 3).

**Table 3:** Lme models for vergence during saccades and fixations.

Predictor	Coefficient	Std. Error	t value
**Summed saccade vergence**			
Monocular cue (intercept)	−0.58	0.1	−5.73***
Normal	0.001	0.06	0.01
Congruent	0.42	0.09	4.84***
Incongruent	−0.31	0.06	−5.24***
Summed saccade duration	−0.04	0.006	−6.24***
**Proportion of saccade convergence**			
Monocular cue (intercept)	−20.02	3.17	−6.33***
Normal	0.34	2.40	0.14
Congruent	15.07	3.37	4.48***
Incongruent	−7.15	2.29	−3.12***
**Summed fixation vergence**			
Monocular cue (intercept)	0.60	0.09	6.54***
Normal	−0.05	0.06	−0.91
Congruent	0.37	0.08	4.70***
Incongruent	−0.50	0.06	−9.14***
Summed fixation duration	−0.0003	0.00003	9.15***
**Proportion of fixation convergence**			
Monocular cue (intercept)	21.73	3.06	7.10***
Normal	−4.30	2.05	−2.09*
Congruent	−0.17	2.89	−0.06
Incongruent	−23.65	1.97	−12.02***

All variables except experimental condition were centered and proportions were arcsine transformed.

* *p*<0.05;

** *p*<0.01;

*** *p*<0.005.

For the analyses of the proportion of saccades/fixations per trial during which convergence (as opposed to divergence) occurred, values were arcsine transformed (to account for the fact that proportion data are non-normally distributed) before being entered into the model. For these analyses, each saccade and fixation was classified as being either convergent (in which case the point of fixation moved closer to the subject) or divergent (in which case the point of fixation moved further away from the subject). Those that did not contain any detectable vergence (2.3% of saccades and 3.5% of fixations) were excluded from the analyses. We analyzed the cases in which a convergent movement was observed, and the intercept was compared to 50% (if convergence and divergence were equally likely to occur, then the proportion of convergence would not be significantly different from 50%; any significant differences indicate a difference in the relative proportions of convergence and divergence, with one being more likely than the other). Given that the analysis is based on the relative proportions of convergence and divergence, and that each saccade/fixation was classified as falling into one of these two categories, then proportions of convergence and divergence are dependent – if one increases, then the other must decrease. For this reason, we analyzed only one of the two categories (the proportion of convergence). Obviously, the effects in the convergence analyses necessarily hold true for the divergence analyses (albeit in the opposite direction).

For summed saccade vergence we entered summed saccade duration as a predictor in the analysis, which produced a significant, negative effect on saccade vergence (*t* = −6.24, *p*<.001), indicating that, on trials with longer saccades there was more divergence exhibited during saccades. This may appear to contrast with the start of fixation disparity analyses where saccade amplitude (which is highly correlated with saccade duration; [1]) was not a significant predictor, but one must bear in mind that, since the dependent measure is aggregated across a trial, location in the sentence is no longer a predictor in the model. We accounted for a lack of an effect of saccade amplitude in the previous analyses because the variability associated with saccade amplitude was greater accounted for by distance through the sentence. Without distance through the sentence in the current analysis, we now see saccade amplitude having a predictive effect on vergence.

As expected, in the monocular cue condition, the eyes tended to diverge during saccades (−0.51°; summed saccade vergence was significantly different from 0; *t* = 5.73, *p*<.001). Summed saccade vergence in the normal condition was not significantly different from the monocular cue condition, showing that the eyes also diverged during saccades (−0.55°; *t*<1). In contrast, in the congruent condition (where the sentence appeared to be looming out from the screen) summed saccade vergence was significantly greater than in the monocular condition–there was significantly less divergence during each trial (−0.45°; *t* = 4.84, *p*<.001). The reduced divergence appears to have occurred as a result of the disparity cue suggesting that the sentence was looming towards the reader, in which case the subject should make more convergent movements. In line with this suggestion, in the incongruent condition (where disparity cues suggested that the sentence was receding behind the screen) summed vergence was significantly smaller than in the monocular condition–there was significantly more divergence during each trial (−0.90°; *t* = 5.24, *p*<.001). Again, it appears that disparity impacted the amount and nature of vergence observed.

With respect to our analysis of the proportion of saccade convergence (as opposed to divergence), in the monocular cue condition we found that convergence occurred in significantly fewer than half of the saccades made (i.e., in the majority of saccades, the eyes diverged; an overall convergent change during the saccades in a trial occurred during only 25% of trials; significantly different from 50%; *t* = 6.33, *p*<.001). The normal condition did not significantly differ from the monocular cue condition in the proportion of saccade convergence (27%; *t*<1, *p*>.5). Consistent with our analysis of the summed saccade vergence, the congruent condition had a significantly larger proportion of saccade convergence than the monocular cue condition (30%; *t* = 4.48, *p*<.001) and the incongruent condition had a significantly smaller proportion of saccade convergence (and, therefore, had a significantly larger proportion of divergent saccades; 16% convergent; *t* = −3.12, *p*<.005).

There was a clear response to the disparity cue during saccades. While there was a tendency for the eyes to diverge during a saccade without a disparity cue in the stimulus, there was significantly less or more divergence when a disparity cue indicated that there should be more convergence (i.e., in the congruent condition) or less convergence (i.e., in the incongruent condition) respectively. This was observed in terms of both the likelihood of making a direction-appropriate movement, as well as the magnitude of that movement.

#### Vergence during fixations

For the summed fixation vergence measure we entered summed fixation duration as a predictor in the analysis, which produced a strong positive effect on fixation vergence, with longer fixations exhibiting more convergence (*t* = 9.15, *p*<.001). As with the effect of saccade amplitude on vergence during saccades, it is likely that the significant effect of fixation duration in these analyses (which is not significant in the end of fixation disparity analyses) is due to the fact that location through the sentence is not a predictor entered in the model. In the monocular cue condition, the eyes produced a significant amount of convergence during fixations (.57°; summed fixation vergence was significantly different from 0; *t* = 6.54, *p*<.001). Summed fixation vergence in the normal condition was not significantly different from the monocular cue condition (.55°; *p*>.05). In the congruent condition summed fixation vergence was significantly greater than in the monocular cue condition (1.03°; *t* = 4.70, *p*<.001) and in the incongruent condition it was significantly smaller than in the monocular cue condition (.13°; *t* = 9.14, *p*<.001).

As in our analysis of vergence during saccades, all fixations were classed as being convergent or divergent, and we then analysed the proportion of fixation convergence compared to 50%. There was a significant proportion of fixations that exhibited convergence in the monocular cue condition (79%; significantly different from 50%; *t* = 7.10, *p*<.001). In the normal condition there was a significantly smaller proportion of fixation convergence than in the monocular cue condition (73%; *t* = 2.09, *p*<.05). This difference is consistent with our analyses of fixation disparity, showing a tendency for larger uncrossed fixation disparities in the monocular condition compared to the normal condition. The congruent condition did not differ from the monocular cue condition (although this is the contrast with less power to detect a real effect and numerically the proportion of convergent fixations was higher: 85%; *p*>.05) but the incongruent condition had a significantly smaller proportion of fixation convergence (48%; *t* = −12.02, *p*<.001). Thus it seems as if the eyes tend to converge during fixations except in the incongruent condition (when the eyes often diverge, presumably to fuse the stimulus because of the stereoscopic disparity manipulation).

Taking these two sets of analyses together (vergence during fixations and during saccades), it seems as if the disparity cue in the stimuli that we used was primarily responsible for changing the vergence response. While the eyes tend to diverge during saccades under normal non-disparate viewing conditions they do so more when disparity cues indicate divergence is necessary and do so less when disparity cues indicate that convergence is necessary. Consequently, under normal reading conditions, the eyes tend to compensate for the divergence during saccades by converging during fixations. Furthermore, when disparity cues indicate that convergence is necessary the eyes converge more than under normal circumstances (i.e., presumably due to additional convergence that facilitates fusion of the stimulus) and when disparity cues indicate that divergence is necessary the eyes converge less than under normal circumstances.

As can be seen in Figure 4, the pattern of data seen within fixation analyses qualitatively compared with those seen in saccade analyses suggests the following. The eyes tend to be slightly uncrossed in the monocular cue and normal conditions (vergence lines represented in the bottom graph are mostly negative), and the degree of this uncrossed disparity does not change much throughout the sentence. In these conditions, the eyes tend to diverge (the degree of disparity rapidly becomes more negative) during saccades and tend to converge (the degree of disparity slowly moves toward zero) during fixations to compensate for this. In contrast, in the congruent and incongruent conditions (i.e., those with a stereoscopic disparity manipulation) the degree of disparity increases throughout the sentence. To accommodate the change in stereoscopic disparity, in the congruent condition the eyes become more converged (the degree of disparity becomes more positive) and in the incongruent condition the eyes become more diverged (the degree of disparity becomes more negative).

## Discussion

The present data suggest that readers easily coordinate their eyes to fuse the percept of a sentence presented in depth in order to read it for comprehension. The results of the comprehension accuracy and global monocular reading measures reveal that, for the most part, readers did not have more difficulty reading sentences when they contained a disparity depth manipulation than when they did not. However, when the disparity and monocular depth cues conflicted, a situation that readers almost never encounter, readers incurred difficulty, reflected in longer average fixation durations. These data suggest that, on each fixation, readers spent more time but otherwise reading behavior was not affected. Thus, when readers encounter sentences with either no or normal depth cues, reading progresses unaffected. Under all experimental conditions, readers were able to process the sentences such that they could respond accurately to comprehension questions.

In terms of a binocular response to apparent depth, eye movements seem to primarily be driven by disparity depth cues, obtained in both foveal and parafoveal vision. First, the vergence response exhibited in response to the stereoscopic manipulation was compatible with the disparity depth cue (stereoscopic offset), even when that cue conflicted with a monocular depth cue (increasing retinal size). Second, these vergence responses occurred not only during fixations, but also during saccades and therefore must be responding to disparity detected in the parafovea. These data differ from those found by Blythe et al. [25] in which vergence responses to isolated words presented at various eccentricities were only observed once the stimulus was fixated (i.e., appeared in the fovea). However, note that our experimental conditions differ in that the disparity cues were present consistently throughout the entire stimulus and led to highly predictable changes in disparity from fixation to fixation. This was not the case in the Blythe et al. [25] study where disparity magnitude changed randomly from trial to trial. Additionally, these vergence movements occurred on the majority of fixations and saccades during reading of the whole sentence. It is important to note, that the nature of the blocked design (all sentences in one condition were experienced in sequence before the next condition) may have contributed to the large influence of disparity cues. As noted in the introduction, the influence of disparity cues increases with practice [26].

Figures 2 and 3 clearly show that although the direction of fixation disparity (as measured at the depth of the screen) was determined by the direction of the stereoscopic disparity manipulation within the stimuli, participants’ eyes tended not to be perfectly aligned at the apparent depth of the stimulus (indicated by the differences between the red and blue lines in these Figures). Rather, the magnitude of fixation disparity was less than the magnitude of the manipulation and so participants’ eyes became aligned at an intermediate depth between the display screen and the apparent stimulus. This is unsurprising, given that for such stereoscopic manipulations there is a conflict within the stimulus between disparity cues (indicating some change in depth) and blur cues (indicating that the stimulus is at the depth of the screen). The vergence and accommodative systems are, typically very tightly linked in their responses, and dissociations between such cues in stereoscopic displays have been shown to impact visual behavior [15]–[16], [34]–[35].

As shown in Figures 2 and 3, toward the end of the sentence, variability in disparity response occurs across observations. That is, some fixations show a high amount of disparity and others show almost none. This spread in the distribution causes the regression line fit to the data to deviate from the line representing the expected disparity if subjects were fusing the sentence perfectly. It is likely that this spread of variability is due to the increasing retinal size manipulation that, as discussed above, would allow for greater disparities (measured in degree of visual angle) to still fixate the same location (measured in characters). However, it is also possible that this variability is caused by fixations on portions of the sentence where vergence would have been very difficult for the subjects, and potentially, the words would have been tricky to fuse. On these fixations, the subjects may have reverted to fixating the plane of the screen, which would have resulted in a near-zero disparity measure. Note that almost all of the disparities observed were of a smaller magnitude than would be required by the stimulus (i.e., almost all points in the figure are between the red line and the black line). Therefore, while it appears that subjects make vergence movements to some extent, for some larger disparities an appropriate vergence response may not occur either because (a) more disparity is tolerable with larger word object sizes, (b) these disparities are too great to be fused easily and the system halts the response, or (c) readers may be able to achieve fusion (presumably through psychological processing rather than physical alignment of the eyes) without the necessity for a full vergence response.

The present study is important in that it suggests that the vast amount of knowledge we have gained over the past few decades about eye movements during reading (obtained only in two-dimensional reading situations) may be, for the most part, generalized to three-dimensional reading situations. As mentioned before, there are many situations in which the text changes its distance from the reader as the sentence progresses. We can now make inferences about these reading situations, based on data from standard reading studies, because the present experiment suggests that readers (a) do not incur more difficulty reading three dimensional text than two dimensional text, (b) make vergence responses to stereoscopic depth easily and immediately when cues are consistent, and (c) incur negative consequences in the presence of conflicting depth cues (although these consequences are adjusted for fairly easily). In short, the visual and eye movement systems are quite capable of accommodating variable and even conflicting visual input for the cognitive and linguistic processes necessary for reading to proceed almost unaffected.

## References

[1] Rayner, K (1998). Eye movements in reading and information processing: 20 years of research.. Psychol Bull.

[2] Rayner, K (2009). Eye movements and attention in reading, scene perception, and visual search.. Q J Exp Psychol.

[3] Liversedge, SP, Findlay, JM (2000). Saccadic eye movements and cognition.. Trends Cogn Sci.

[4] Blythe, HI, Liversedge, SP, Joseph, HSSL, White, SJ, Findlay, JM (2006). The binocular coordination of eye movements during reading in children and adults.. Vision Res.

[5] Hendriks, AW (1996). Vergence eye movements during fixations in reading.. Acta Psychol.

[6] Juhasz, BJ, Liversedge, SP, White, SJ, Rayner, K (2006). Binocular coordination of the eyes during reading: Word frequency and case alternation affect fixation duration but not binocular disparity.. Q J Exp Psychol.

[7] Kirkby, JA, Webster, LAD, Blythe, HI, Liversedge, SP (2008). Binocular coordination during reading and non-reading tasks.. Psychol Bull.

[8] Liversedge SP, Rayner K, White SJ, Findlay JM, McSorley E (2006). Binocular coordination of the eyes during reading.. Curr Biol.

[9] Liversedge SP, White SJ, Findlay JM, Rayner K (2006). Binocular coordination of eye movements during reading.. Vision Res.

[10] Nuthmann A, Kliegl R (2009). An examination of binocular reading fixations based on sentence corpus data.. J Vis.

[11] Collewijn H, Erkelens CJ, Steinman RM (1988). Binocular coordination of human horizontal saccadic eye movements.. J Physiol.

[12] Collewijn H, Erkelens CJ, Steinman RM (1995). Voluntary binocular gaze-shifts in the plane of regard: Dynamics of version and vergence.. Vision Res.

[13] Collewijn H, Erkelens CJ, Steinman RM (1997). Trajectories of the human binocular fixation point during conjugate and non-conjugate gaze-shifts.. Vision Res.

[14] Erkelens CJ, Sloot OB (1995). Initial directions and landing positions of binocular saccades.. Vision Res.

[15] Hoffman DM, Girshick AR, Akeley K, Banks MS (2008). Vergence-accommodation conflicts hinder visual performance and cause visual fatigue.. J Vis.

[16] Kenyon RV, Ciuffreda KJ, Stark L (1977). Binocular eye movements during accommodative vergence.. Vision Res.

[17] Ono H, Nakamizo S, Steinbach MJ (1978). Nonadditivity of vergence and saccadic eye movement.. Vision Res.

[18] Schor C (1999). The influence of interactions between accommodation and convergence on the lag of accommodation.. Ophthalmic Physiol Opt.

[19] Sylvestre PA, Galiana HL, Cullen KE (2002). Conjugate and vergence oscillations during saccades and gaze shifts: implications for integrated control of binocular movement.. J Neurophysiol.

[20] Zee DS, Fitzgibbon EJ, Optican LM (1992). Saccade-vergence interactions in humans.. J Neurophysiol.

[21] Kirkby JA, Blythe HI, Benson V, Liversedge SP (2010). Binocular coordination during scanning of simple dot stimuli.. Vision Res.

[22] Busettini C, Fitzgibbon EJ, Miles FA (2001). Short latency disparity vergence in humans.. Journal Neurophysiol.

[23] Bussetini C, Miles FA, Krauzlis RJ (1996). Short latency disparity vergence responses and their dependence on a prior saccadic eye movement.. Journal Neurophysiol.

[24] Popple AV, Smallman HS, Findlay JM (1998). The area of spatial integration for initial horizontal disparity vergence.. Vision Res.

[25] Blythe HI, Liversedge SP, Findlay JM (2010). The effective fusional range for words in a natural viewing situation.. Vision Res.

[26] Eggert T, Kapoula Z (1995). Position dependency of rapidly induced saccade disconjugacy.. Vision Res.

[27] Schotter ER, Angele B, Rayner K (2012). Parafoveal processing in reading.. Atten Percept Psychophys.

[28] Rayner K (1979). Eye guidance in reading: Fixation locations within words.. Perception.

[29] van Ee R (2005). Dynamics of perceptual bi-stability for stereoscopic slant rivalry and a comparison with grating, house-face, and Necker cube rivalry.. Vision Res.

[30] Knapen T, van Ee R (2006). Slant perception, and its voluntary control, do not govern the slant aftereffect: Multiple slant signals adapt independently.. Vision Res.

[31] Allison RS, Howard IP (2000). Temporal dependencies in resolving monocularand binocular cue conflict in slant perception.. Vision Res.

[32] Deubel H, Bridgeman B (1995). Fourth Purkinje image signals reveal lens deviations and retinal image distortions during saccadic eye movements.. Vision Res.

[33] Kirkby JA, Blythe HI, Drieghe D, Livesedge SP (2011). Reading text increases binocular disparity in dyslexic children.. PLoS ONE.

[34] Blythe HI, Holliman NS, Jainta S, Tbaily LW, Liversedge SP (invited resubmission)..

[35] Mon-Williams M, Wann JP (1998). Binocular virtual reality displays: When problems do and don’t occur.. Hum Factors: 40,.

